# Influence of particle size, shape, and magnetic properties on torque-driven biofilm removal using anisotropic magnetic particles

**DOI:** 10.1039/d6nr00046k

**Published:** 2026-03-27

**Authors:** Vincent Pautu, Laurine Marger, Maja Caf, Fabrice Marger, Mustapha Mekki, Slavko Kralj, Irena Milošević

**Affiliations:** a School of Engineering, Architecture and Landscape of Geneva (HEPIA), HES-SO – University of Applied Sciences and Arts Western Switzerland Switzerland irena.milosevic@hesge.ch; b Laboratory of Biomaterials, Faculty of Medicine, University Clinics of Dental Medicine, University of Geneva Genève Switzerland; c Department for Materials Synthesis, Jožef Stefan Institute Jamova 39 Ljubljana Slovenia slavko.kralj@ijs.si; d Faculty of Pharmacy, University of Ljubljana Aškerčeva 7 Ljubljana Slovenia

## Abstract

Biofilms are structured communities of bacteria embedded within an extracellular polymeric substance (EPS) matrix, which forms a protective barrier that restricts drug penetration and increases antibiotic tolerance, making their complete elimination particularly challenging. Here, we investigate a magneto-mechanical approach using rotating magnetic fields (RMFs) to deliver controlled mechanical stress to *Enterococcus faecalis* biofilms *via* anisotropic magnetic particles (AMPs). Microrods, nanochains, and nanorods with distinct sizes and magnetic properties were actuated under identical RMF conditions on implant-relevant titanium substrates. Micron-scale magnetic microrods generate sufficient magnetic torque to mechanically disrupt the EPS matrix and detach biofilm structures, significantly increasing suspended bacterial cells without marked bactericidal effects. In contrast, nanoscale AMPs do not induce biofilm detachment but cause membrane damage, increasing the proportion of injured cells. These findings demonstrate a size-dependent transition between microscale biofilm detachment and nanoscale membrane interactions, identifying particle size as the dominant parameter governing magneto-mechanical biofilm disruption.

## Abstract 2

Using rotating magnetic fields and different anisotropic magnetic particles to deliver controlled mechanical stress, persistent *Enterococcus faecalis* biofilms were disrupted.

## Graphical abstract

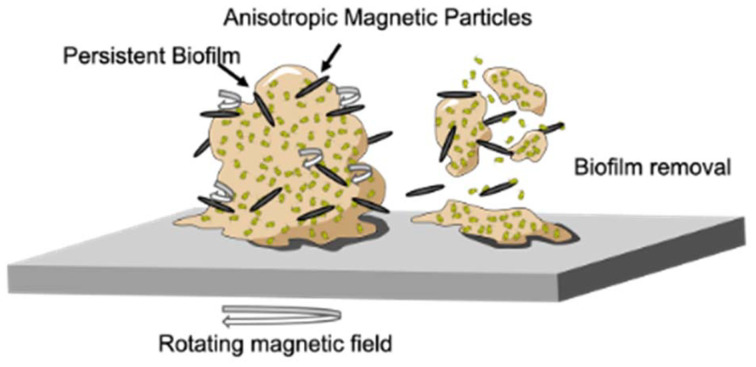

## Introduction

Biofilms are complex structured communities constituted of micro-colonies of bacterial cells that are embedded within a self-produced extracellular polymeric substance (EPS) matrix, which adheres to both biological and artificial surfaces.^1^ The EPS matrix is mainly composed of water (up to 97%), functioning as a solvent for essential structural and functional components, including soluble gel-forming polysaccharides, proteins, and extracellular deoxyribonucleic acid (DNA). Moreover, EPSs control adhesion, mechanical stability, diffusion, and stress dissipation at solid–liquid interfaces.^2,3^ Biofilms can irreversibly adhere to a wide range of surfaces, both biotic (such as tissues) and abiotic (such as medical devices, industrial materials, or water pipes).

Once established, biofilms create a barrier that protects bacteria from conventional antimicrobial therapies by restricting drug penetration, increasing antibiotic tolerance, and facilitating intercellular communication, which increases resistance and diminishes the efficacy of treatment against biofilms.^4^ This resistance results in persistent infections and complicates both preventive and therapeutic strategies, as resistance to antibiotics is augmented by approximately 1000-fold.^5^ The biofilm matrix is not only a biochemical barrier but also a mechanical barrier with spatially varying stiffness and relaxation times. This barrier gives biofilms their characteristic resilience and tolerance to shear, which can be even more pronounced when grown under shear conditions.^6–8^ As an example, scaling and root planing are employed to eliminate plaque and biofilm; however, these mechanical procedures, involving scraping and smoothing teeth roots, are often inadequate for the complete eradication and enduring elimination of all microorganisms.^9^ Among them, *Enterococcus faecalis* (*E. faecalis*) is frequently detected in persistent endodontic and *peri*–implant infections and is well known for its ability to survive conventional mechanical debridement and irrigation procedures. Its capacity to form highly resilient biofilms, combined with tolerance to disinfectants and antibiotics, makes *E. faecalis* a likely contributor to residual biofilm and reinfection around dental and implant surfaces.^10^

The adhesion strengths underline why physical removal or preventive surface modification is critical. Such strong bacterial adhesion is a primary reason why titanium implants are vulnerable to *peri*-implant infections, necessitating advanced anti-biofilm surface technologies or nanoparticle-based approaches for long-term protection and easy cleaning.^11^

From a materials perspective, the problem caused by biofilms is primarily mechanical rather than biological: how can one apply a controlled stress strong enough to fragment or detach such a structure without relying solely on chemical agents? This mechanical challenge is compounded by the fact that the EPS matrix acts as a dense viscoelastic protective barrier, severely limiting the penetration of antimicrobial agents into the encapsulated bacterial cells.

Addressing this dual challenge, both physical disruption and enhanced penetration, has led to the development of various strategies to disrupt the biofilm structure and improve the delivery of antibacterial agents. Among these approaches, the use of magnetic nanoparticles has emerged as a promising tool due to their ability to disrupt the biofilm matrix and enhance antibiotic delivery. Iron oxide nanoparticles (IONPs) under a static magnetic field used in conjunction with antibiotics demonstrate significant efficacy in eradicating bacteria within the biofilm.^12,13^ These studies used the magnetic responsiveness of spherical nanoparticles to facilitate the formation of channels and penetration inside the biofilm matrix and consequently enhance the accessibility of antimicrobial agents to the bacterial cells.

However, the central challenge remains: how to inject sufficient mechanical energy into the hydrated EPS network to achieve disruption? Magnetic actuation offers a contactless route to deliver mechanical work and provides one possible solution to deliver controlled energy through rotating magnetic fields (RMFs), which generate a magnetic torque (*τ* = *μ* × *B*, where *τ* is the magnetic torque, *μ* is the magnetic moment and *B* is the magnetic field) on magnetic objects. This torque converts magnetic energy into rotational motion at the micro- or nanoscale and depends on the magnetic moment of the materials and the magnetic field applied. Unlike static magnetic approaches, RMF-driven rotation offers the potential to mechanically disrupt the biofilm structure through continuous physical agitation.

Building upon this principle, more advanced systems rely on magnetic particles with anisotropic shapes, which are able to rotate under exposure to RMFs.^14,15^ The mechanical work delivered by these systems correlates with both magnetic anisotropy and the aspect ratio of the particles. This approach relies on the conversion of magnetic energy into mechanical agitation and creates not only unidirectional channels but also generates rotational mechanical movement that results in biofilm sensitization, enhanced infiltration, and physical disruption due to the magnetic torque generated by the rotating anisotropic magnetic particles (AMPs). In essence, AMPs combine the penetration-enhancing benefits of spherical magnetic nanoparticles with the added mechanical disruption capability of sustained rotational motion, offering a multifaceted strategy for biofilm eradication.^16,17^

In this work, various AMP objects, named nanorods, nanochains, and microrods, were prepared based on (1) the magnetic assembly of IONPs obtained through a modified Massart's coprecipitation method^18^ or (2) the chemical reduction of an akageneite precursor.^19^ The influence of such AMPs differing in their aspect ratios, size and magnetic characteristics and subjected to a RMF was studied on an *E. faecalis* biofilm. The bacterial detachment from the biofilm was analysed using flow cytometry, allowing us to compare the biofilm-disrupting efficiency of the different AMP types.

## Experimental

### Chemicals

The chemicals used for the syntheses of iron oxide nanoparticles were of reagent grade quality and were obtained commercially. Iron III chloride hexahydrate (FeCl_3_·6H_2_O, ≥99%, CAS 10025-77-1), dopamine hydrochloride (C_8_H_11_NO_2_·HCl, 99%, CAS 62-31-7), oleylamine (C_18_H_37_N, 80–90%, CAS: 112-90-3), and tetraethyl orthosilicate (Si(OCH_2_CH_3_)_4_, 98% CAS 78-10-4) were purchased from Brunschwig. Ammonium hydroxide solution (NH_3_, 25 wt%, CAS 1336-21-6), hydrochloric acid (HCl, ≥32%, CAS: 7647-01-0), and absolute ethanol of analytical reagent grade (EtOH, ≥99.8% CAS: 64-1-5) were purchased from Carl Roth. Iron(iii) sulphate hydrate, iron(ii) sulphate heptahydrate (ACS, 99%), citric acid (CA, 99%), acetone (99.5%), and NH_4_OH (25%) were supplied by VWR Int. GmbH (Vienna, Austria). Ethanol (absolute reagent; 99.5%) was purchased from Carlo Erba Reagents (Milan, Italy). Ricinoleic acid (>80%) was purchased from TCI Chemicals. Dichloromethane (DCM) and dimethylformamide (DMF) were obtained from Sigma-Aldrich (St Louis, MO, USA). Sodium chloride (≥99.5%) was obtained from Fisher Scientific. All the chemicals were used as purchased without further purification. Ultrapure Millipore water (18.2 MΩ) was used throughout the entire study. All glassware was washed with aqua regia and rinsed with abundant water before use.

### Synthesis of anisotropic magnetic particles

#### Synthesis of superparamagnetic microrods (MMRs)

To obtain MMRs, the first step involved the synthesis of hydrophilic iron oxide nanocrystals, which served as the building blocks for microrod formation. These nanocrystals were prepared by co-precipitation of Fe^2+^ and Fe^3+^ ions from an aqueous solution according to previously published protocols.^20^ Briefly, the iron(ii) and iron(iii) salts were dissolved to final concentrations of 27 mmol L^−1^ Fe^2+^ and 14 mmol L^−1^ Fe^3+^. The precipitation of iron oxide nanocrystals was triggered using ammonia (∼25%, aqueous) in two steps. First, the pH value was adjusted to 3 and kept constant for 30 min. Then, the pH value was increased to 11.6. After an additional aging for 0.5 hours, the formed hydrophilic iron oxide nanocrystals were collected using a permanent NdFeB magnet (Supermagnete GmbH, Gottmadingen, Germany), washed three times with diluted aqueous ammonia solution at pH 10.5, and then finally dispersed in water (120 mL). The washed hydrophilic iron oxide nanocrystals were further processed with citric acid attachment.^21^ Here, a CA aqueous solution (5 mL, 0.5 g mL^−1^) was added to the suspension of nanocrystals in water (120 mL), and the pH value was adjusted to 5.2 with ammonia solution (∼25%, aqueous). The reaction mixture was stirred in an oil bath at 80 °C for 90 min. The pH value was then increased to 10.2 using ammonia solution (∼25%, aqueous). Finally, the obtained suspension was centrifuged at 5000*g* for 5 min to remove possible aggregated iron oxide nanocrystals while the supernatant, representing the ferrofluid, was used for further procedures.^22^

The MMRs were then produced by magnetic assembly of citric acid-coated nanoparticles (MNP-CA), followed by silica deposition for structural fixation.^23^ First, an aqueous suspension of MNP-CA nanocrystals (75 mL) and, separately, a 0.172 M tetraethyl orthosilicate (TEOS) solution in absolute ethanol were prepared. The nanoparticle suspension was stirred at 400 rpm for 2 minutes, followed by the addition of 1.5 mL of 25% aqueous ammonia to initiate the sol–gel process. The TEOS solution was then added dropwise under continuous stirring. After approximately 1 minute, 3.0 mL of a saturated NaCl aqueous solution was introduced to enhance rod formation through ionic strength modulation. The mixture was stirred for an additional 2 minutes and subsequently exposed to an external magnetic field (24 mT) and left undisturbed overnight to facilitate magnetic alignment and silica condensation. The resulting microrods were collected and purified by magnetic separation and repeated washing with deionized water to remove unreacted components and excess MNP-CA.

To improve the structural stability of the anisotropic microrods, an additional thin silica shell was deposited. The previously synthesized magnetic microrods were dispersed in a solvent mixture of 100 mL of deionized water and 250 mL of absolute ethanol. In parallel, 17 mL of a 0.790 M TEOS solution in ethanol was prepared. To initiate the silica coating process, 1.65 mL of 25% aqueous ammonia (NH_4_OH) was added to the microrod suspension, followed by the gradual addition of the TEOS solution under continuous stirring at 250 rpm. To ensure sustained hydrolysis and condensation, additional two aliquots of NH_4_OH (1.65 mL each) were added at one-hour intervals during the reaction. The coating process was allowed to proceed for a total of four hours. Upon completion, the silica-coated microrods were collected and purified by repeated magnetic separation and washing with deionized water to remove any unreacted precursors and by-products.

#### Synthesis of superparamagnetic nanochains (MNCs)

Magnetic nanochains were synthesized according to previously established protocols.^16,18,24^ In brief, the process began with the self-assembly of individual iron oxide nanocrystals into spherical nanocrystal clusters, approximately 100 nm in diameter, as detailed in earlier work.^25–27^ These nanocrystal clusters were then magnetically aligned into linear chain-like structures using a magnetic templating technique, following the method described in the work of S. Kralj and D. Makovec.^18^ To preserve and stabilize the anisotropic morphology, the dynamically formed nanochains were subsequently stabilized with a uniform silica shell, as outlined in our previously published studies.^28,29^

#### Synthesis of superparamagnetic nanorods (MNRs)

Hydrophobic iron oxide nanocrystals serve as building blocks for the synthesis of MNRs and they are obtained through co-precipitation of Fe^2+^ and Fe^3+^ salts using aqueous ammonia as published elsewhere.^30^ Specifically, 400 mL of aqueous ammonia was added to 500 mL of a freshly prepared solution containing Fe^2+^ (0.135 mol L^−1^) and Fe^3+^ (0.113 mol L^−1^) ions under vigorous stirring at 75 °C, increasing the pH to above 9.5. To hydrophobize the nanocrystals, 10 g of RA was added incrementally over a period of 30 minutes immediately following the addition of aqueous ammonia. The resulting suspension was aged at 80 °C for an additional 30 minutes. After cooling to room temperature, diluted nitric acid (pH 1) was added dropwise to adjust the pH to approximately 5, inducing flocculation of the hydrophobic iron oxide nanocrystals. To remove unreacted ions and excess RA, the precipitate was washed three times with deionized water and methanol. The RA-functionalized nanoparticles were then dried and redispersed in chloroform for storage.

For MNR synthesis, the hydrophobic nanoparticles were dispersed in dichloromethane to a final volume of 5 mL (5 mg mL^−1^). In parallel, a solution of 20 mL of deionized water and 80 mL of absolute ethanol was prepared in a beaker positioned within a 37 mT magnetic field. A mechanical stirrer was introduced and set to operate at 500 rpm. To initiate the synthesis, 2.5 mL of 25% aqueous ammonia (NH_4_OH) was added to the ethanol–water mixture. The hydrophobic iron oxide nanocrystal suspension was then introduced dropwise into the vigorously stirred solution. After 5 minutes of continuous stirring, the external magnetic field was removed and 200 µL of tetraethyl orthosilicate (TEOS) was added dropwise. The reaction mixture was stirred for an additional 30 seconds and subsequently left undisturbed for 3 hours to allow for silica condensation. The resulting MNRs were collected by magnetic separation and purified through three washing cycles with absolute ethanol, followed by three washes with deionized water.

#### Synthesis of ferromagnetic nanorods (FMNRs)

The synthesis method was adapted from previous work;^19^ in a typical procedure, 180 mL of ultrapure water was heated to 80–90 °C. Then, 1 mL of dopamine hydrochloride solution (3.2 mg mL^−1^) and 800 μL of 1 M HCl were added under stirring. Subsequently, 20 mL of FeCl_3_·6H_2_O solution (1 mol L^−1^) was added to the mixture. The reaction was maintained at 80–90 °C under stirring for 6 hours. After this time, the mixture was allowed to cool naturally to room temperature. The product was collected by centrifugation at 7690*g* for 15 minutes and washed three times with ultrapure water. Finally, the particles were redispersed in ultrapure water. An orange colloidal suspension of β-FeOOH nanorods was obtained.

The silica coating of β-FeOOH nanorods was performed following a modified Stöber process, adapted from a previously published protocol.^31^ Briefly, 100 mg of β-FeOOH nanorods were dispersed in a mixture consisting of 140 mL of absolute ethanol, water (15 mL final concentration), and 10 mL of ammonium hydroxide solution (25 wt%). The suspension was stirred for 5 min at room temperature to ensure homogeneous dispersion. Subsequently, 5 μL of tetraethyl orthosilicate (TEOS), pre-diluted in 10 mL of ethanol, was added dropwise to the mixture under vigorous stirring. The reaction was allowed to proceed for 2 h at room temperature to form a uniform silica shell around the nanorods. The resulting SiO_2_@β-FeOOH nanorods were collected by centrifugation at 7600*g* for 5 min, washed three times with ethanol, and redispersed in 10 mL of ethanol to obtain a final concentration of 10 mg mL^−1^.

A total of 100 mg of SiO_2_@β-FeOOH nanorods was recovered by centrifugation at 7600*g* for 10 min to remove the ethanol supernatant. The pellet was redispersed in 20 mL of oleylamine and transferred into a microwave reaction vessel. Argon gas was bubbled through the mixture for 5 min to eliminate dissolved oxygen and air from the vessel. The vessel was then sealed and heated to 200 °C for 2 h under microwave irradiation.

After cooling to room temperature, the resulting black product was recovered by magnetic separation, washed six times with a mixture of ethanol and isopropanol, followed by three washes with ultrapure water. The final product was redispersed in 5 mL of ultrapure water.

### Titanium substrates

Commercially pure titanium grade 4 (Ti G4) plates (10 × 10 × 1 mm^3^) were obtained from Bibus Métal AG (Fehraltorf, Switzerland). The plates were produced by industrial cold rolling (laminated finish, not mirror-polished) and were accompanied by manufacturer's certificates of conformity in compliance with ASTM F67/ISO 5832-2. Handling was performed with nitrile gloves to prevent surface contamination.

Before characterization, each plate was subjected to a standardized cleaning sequence: (1) ultrasonic cleaning in a 3% (v/v) titanium-specific alkaline detergent (Galvex® 17.3%, NGL Cleaning Technology, Switzerland) in deionized water for 10 min; (2) double rinse in deionized water (2 × 2 min); (3) rinse in isopropanol (≥99.5%) for 2 min; and (4) drying with filtered compressed air (Class 100). After cleaning, the plates were sterilized by saturated-steam autoclaving at 121 °C for 20 min and stored in heat-sealed sterile pouches until use.

### Characterization

#### Surface topography and roughness measurement

The surface roughness was quantified using a CT100 non-contact confocal laser profilometer (Cyber Technologies GmbH, Germany). Five randomly selected positions per plate were recorded over 5 mm scan lengths. A Gaussian filter (cut-off *λ*_c_ = 0.8 mm) was applied following ISO 4287 and ISO 16610-21 to separate roughness from waviness. The arithmetic mean roughness (*R*_a_) and complementary amplitude parameters (*R*_q_, *R*_c_, and *R*_z_) were calculated directly from raw profiles. All values are reported as mean ± standard deviation (*n* = 9).

#### Surface wettability

Static water contact-angle measurements were performed using an Attension theta optical tensiometer (Biolin Scientific, Finland). Measurements were carried out under laminar flow at 22 ± 2 °C using 5 µL ultrapure-water droplets deposited automatically on the surface. Contact angles were determined after 10 s using the Young–Laplace fitting model. Tree droplets were analyzed per specimen (*n* = 5 plates), and the results are expressed as mean ± standard deviation.

#### Atomic absorption spectroscopy (AAS)

The concentration of Fe ions was determined using atomic absorption spectroscopy with a PinAAcle 500 PerkinElmer AAS in combination with Syngistix v5.1.0. In a standard procedure, particles were destroyed in 50% aqua regia. The resulting solution was then analyzed by AAS to determine the iron concentration.

#### Transmission electron microscopy (TEM) analysis

The transmission electron microscopy analyses were performed using a transmission electron microscope (TEM, Jeol 2100, Akishima, Japan) coupled with energy-dispersive X-ray spectroscopy (EDXS, JED 2300 EDS). For all nanoparticle types, the samples were deposited on TEM grids (200 mesh; SPI Supplies, West Chester, PA) by placing a few drops of the diluted sample and then allowing them to dry. The size distribution was analysed using ImageJ (version 1.53t, Java 1.8.0), with a sample size of *N* = 200. The form ratio was calculated as follows: length/width.

#### Vibrating-sample magnetometry

Magnetic measurements were carried out at room temperature using a vibrating-sample magnetometer (VSM 7307; Lake Shore Cryotronics, Westerville, OH) with a maximum applied magnetic field of 10 kOe. A measured mass of the dried, ground sample (10–20 mg) was compressed into the sample holder and positioned within the uniform magnetic field of the VSM system.

#### Rotating magnetic field

The magnetic field produced by a MIXdrive 24 MTP induction mixer (from 2mag, Munich, Germany) was characterized with a handheld Gauss Tesla meter FH 21 (from Magnet Physik Dr Steingroever GmbH, Germany) equipped with an axial Hall probe. The probe tip was positioned at the bottom center of individual wells of an empty 24 well plate resting on the mixer. For each combination of mixer power and rotation speed, measurements were taken in all 24 wells following the same positioning procedure. These data were used to determine the field distribution across the 24-well plate. The conditions tested matched those used in the biofilm detachment experiments on titanium discs. Numerical values and comparison with previous work are reported in the Results section.

### *E. faecalis* biofilm cultures

*Enterococcus faecalis* (ATCC-29212, University of Geneva) was initially cultured on Brain Heart Infusion (BHI) agar (Becton Dickinson). Single colonies were transferred into 8 mL of BHI broth and incubated aerobically at 37 °C overnight. The bacterial suspension was then centrifuged at 4000*g* for 5 minutes and the optical density was adjusted to OD_600_ = 0.5.

Sterile grade 4 titanium plates (dimensions: 10 mm × 10 mm) were placed in 24-well plates, and each well was inoculated with the standardized *E. faecalis* suspension. The plates were incubated for 24 hours at 37 °C to allow biofilm formation.

### Magnetically actuated treatment

Following the 24-hour biofilm formation, culture media were aspirated and the biofilms were washed three times with PBS. Titanium plates were then exposed to 4 various AMPs: MMRs, MNRs, MNCs and FMNRs. Two controls, one negative (*i.e.*, without AMPs) and one positive (a copper plate), were added for each experiment. The negative control (Ti plate without AMPs) was used to verify that the magnetic field exposure alone does not alter biofilm structure or viability. The positive control (Cu plate) served as a reference for a surface with well-documented antibiofilm and antibacterial properties, providing a benchmark for maximal biofilm reduction under the same conditions.

These AMPs were suspended at defined concentrations in PBS and added to each well.

Magnetic agitation was performed using a rotating magnetic field generated by a 2mag MIXdrive 24 MTP system (Germany) operated at 33 Hz or 2000 rpm at the maximum power (10 W). Instead of the magnetic hyperthermia regime, which relies on high-frequency (>100 kHz) alternating magnetic fields to generate heat,^17^ the present work uses the low frequency regime under a rotating magnetic field to deliver “controlled mechanical stress,” which is far from any heating contribution originating from magnetic nanoparticles. The protocol consisted of three agitation cycles of 3 min each (total agitation time 9 min).

Between each agitation cycle, the suspension of AMPs was briefly resuspended manually with a 200 µL pipette to maintain a homogeneous particle distribution, minimize aggregation, and preserve the effective rod aspect ratio and torque generation.

The necessity and efficiency of this resuspension step were verified in a preliminary optimization experiment using MMRs at 100 ppm (iron concentration). Three conditions were compared under identical RMF parameters:

(i) a control without rods (mechanical resuspension only), (ii) MMRs with manual resuspension between each 3-minute cycle (3 × 3 min + resuspension), and (iii) MMRs without resuspension (continuous 9-minute agitation).

At the end of the agitation, both the planktonic fraction and the bacteria adhered to the titanium surfaces were collected. The discs were vortexed and sonicated as previously described.

### Chlorhexidine treatment of biofilms

*E. faecalis* biofilms were grown for 24 h on titanium discs as described above. The discs were then exposed to 0.1% chlorhexidine (CHX) solution for either 30 s (representing typical dental clinical exposure) or 9 min, corresponding to the total duration used in the magnetic actuated treatment. After treatment, the discs were rinsed three times with PBS to remove residual CHX and non-adherent or dead bacteria.

### Flow cytometry staining procedure

Both the planktonic bacterial suspensions and biofilm-detached bacteria were assessed for membrane integrity using a LIVE/DEAD BacLight Bacterial Viability Kit (Thermo Fisher Scientific) containing SYTO9 and propidium iodide (PI). SYTO9 permeates all bacterial membranes and stains live cells, while PI stains only membrane-compromised (dead or damaged) cells.

The staining solution was prepared by mixing SYTO9 and PI in a 1 : 1 ratio in PBS. Equal volumes of the staining solution and bacterial suspension were mixed and incubated for 15 minutes at room temperature in the dark. Samples were analyzed using a CytoFLEX flow cytometer (Beckman Coulter MoFlo Astrios) equipped with four lasers, counting 10 000 objects.

Excitation/emission settings were SYTO9 (Ex. 488 nm/Em. 504–555 nm) and PI (Ex. 561 nm/Em. 570–675 nm). Samples were acquired at a flow rate of 10 µL min^−1^ for 120 s and analyses were restricted to the bacterial gate (approximately 10^6^ cells per mL). Cytometric gates were established using bacterial reference controls including untreated live cells, ethanol-killed cells (70% EtOH, 10 min), and single-stained controls (SYTO9 only or PI only) to verify channel separation. Based on these controls, three populations were defined live (SYTO9+/PI−), membrane-compromised (“injured”) (SYTO9+/PI+), and dead (SYTO9−/PI+). Results were expressed as the percentages of live, injured, and dead bacterial cells. To quantify bacteria detached by the different AMPs and present in suspension, the number of events per µL was measured for each group and normalized to the control group. All experiments were performed in triplicate.

### Scanning electron microscopy (SEM) analysis

After 24 hours of biofilm formation and subsequent magnetic agitation (3 × 3 minutes) with the different nanorod formulations, the titanium discs were rinsed with PBS and fixed in 2.5% glutaraldehyde in 0.1 M sodium cacodylate buffer for 30 minutes. The discs were then rinsed in PBS and dehydrated through a graded ethanol series (50%, 70%, 90%, and 100%) for 10 minutes each.

Samples were sputter-coated with a 20 nm layer of gold and examined using a Sigma 300 VP FE-SEM (Zeiss, Germany) to visualize the biofilm morphology.

### Statistical analysis

Data normality was assessed using the Shapiro–Wilk test (significance threshold set at *p* < 0.05). As data did not follow a normal distribution, non-parametric statistical tests (Kruskal–Wallis rank test) were applied with Dunn's multiple comparison test. Statistical analyses were conducted using Prism 10 (GraphPad Software Inc., La Jolla, CA), and a significance level of *p* < 0.05 was considered statistically significant.

## Results and discussion

### Rationale for particle design and the experimental system

**Fig. 1:**
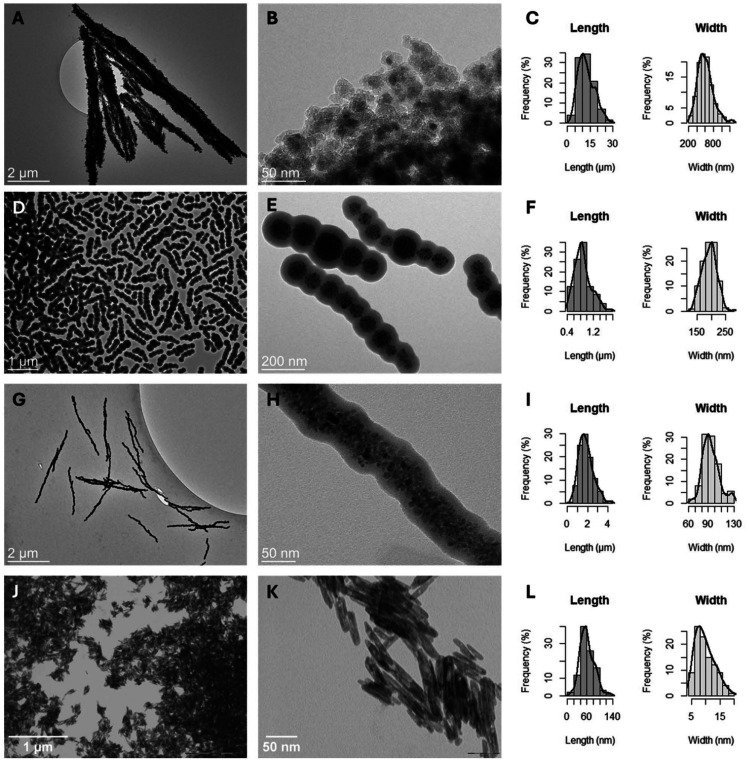
Transmission electron micrographs (TEMs) and size distribution histograms (left: length distribution; right: width distribution) of microrods MMRs (A–C), nanochains MNCs (D–F), nanorods MNRs (G–I) and nanorods FMNRs (J–L). Size distributions were obtained by analyses of TEM images. Frequencies are expressed as a percentage of the total number of measured particles (*n* > 100).

To systematically evaluate magneto-mechanical actuation effects on bacterial biofilms, we engineered four types of anisotropic magnetic particles (AMPs) with distinct dimensions (from nanoscale to microscale lengths), aspect ratios (from 4.6 to 22.8) and magnetic properties (superparamagnetic and ferromagnetic) (Fig. 1 and Table 1). The design strategy was based on the hypothesis that particle size, aspect ratio, and magnetic behavior would differentially influence biofilm penetration, mechanical torque generation, and bactericidal effects under RMFs, thereby enabling a comparative analysis of how these parameters influence the particles’ capacity to interact with biofilms and mechanically disturb or loosen their components under RMF application.

**Table 1:** Characteristics of AMPS

Materials	Length (nm)	Width (nm)	Aspect ratio	Emu g^−1^
MMR	12 600 ± 5200	605 ± 184	22.8 ± 11.6	33.9
MNC	882 ± 255	193 ± 26	4.6 ± 1.5	13.1
MNR	1900 ± 700	94 ± 13	19.9 ± 7.8	15.4
FMNR	61 ± 21	10 ± 2	6.5 ± 2.2	3.0

**Fig. 2:**
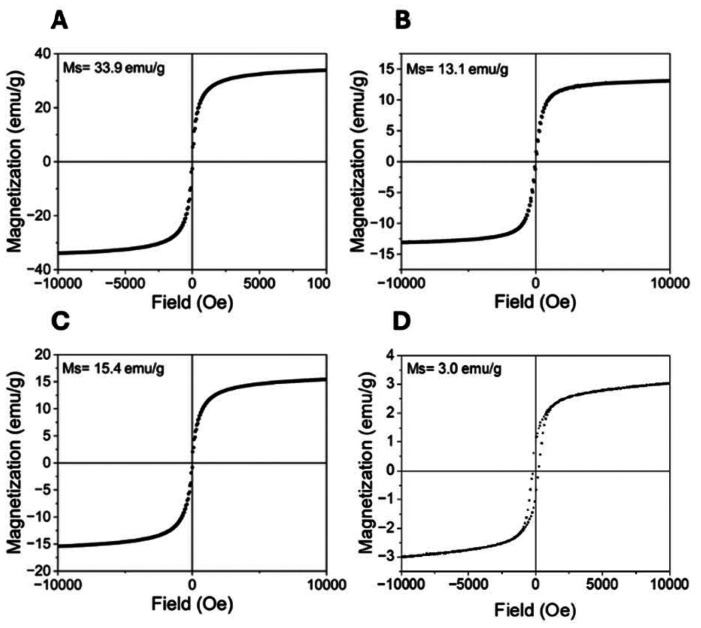
Room-temperature measurements of the mass magnetization as a function of magnetic field for the MMRs (A), MNCs (B), MNRs (C), and FMNRs (D).

MMRs were assembled from citric acid-stabilized iron oxide nanocrystals through magnetic field-directed aggregation, resulting in rod-like structures with an average length of 12.6 ± 5.2 µm and a width of 605 ± 184 nm. These superparamagnetic assemblies exhibited a saturation magnetization of 33.9 emu g^−1^ (Fig. 2A). MNCs were prepared by magnetic assembly of 100 nm-sized nanocrystal clusters into chain-like structures with dimensions of 882 ± 255 nm (length) × 193 ± 26 nm (width) and a saturation magnetization of 13.1 emu g^−1^ (Fig. 2B). MNRs exhibited dimensions of 1900 ± 700 nm × 94 ± 13 nm with a saturation magnetization of 15.4 emu g^−1^ (Fig. 2C). FMNRs, synthesized through a three-step process involving akageneite precursors and organo-thermal reduction, measured 61 ± 21 nm × 6.5 ± 2.2 nm and displayed weak ferromagnetic behavior with a remanent magnetization of 0.9 emu g^−1^ and a saturation magnetization of 3.0 emu g^−1^. All AMPs were stabilized with a silica shell to preserve the morphology during agitation and provide a biocompatible, hydroxyl-rich surface for biofilm interaction, a critical consideration given that particle aggregation or surface chemistry changes could confound mechanical effects with unintended biological interactions.^32^ All the characteristics of the different AMPs are summarized in Table 1 for more clarity.

The use of commercially pure titanium grade 4 substrates with a cold rolled finish provided an implant relevant baseline representative of clinically used titanium surfaces.^11,33,34^ This choice was made to reproduce realistic dental and implant conditions while maintaining a controlled and reproducible substrate.

Surface characterization was performed prior to biofilm experiments. Profilometry confirmed a homogeneous surface topography with a roughness *R*_a_ of 0.82 ± 0.03 µm (see SI Table S1). Static water contact angle measurements indicated moderate hydrophilicity with a value of 79.8 ± 0.8° (see SI Table S2), consistent with the presence of a native titanium dioxide passive layer on titanium surfaces.^11,33^

This characterization documents that all experiments were conducted on comparable substrates. It minimizes substrate-driven variability between samples and supports the interpretation of the magneto mechanical effects as particle dependent rather than surface dependent. Prior to conducting the magneto-mechanical experiments on biofilms, the magnetic field generated by the 2mag MIXdrive was characterized to ensure consistent RMF exposure across all wells (see SI Tables S3 and S4 for magnetic field values at different powers and frequencies). Under the operating conditions used for biofilm experiments (100% power, 33 Hz), the average field strength at the titanium disc level was 8.6 ± 1.5 mT (range 7.13–10.87 mT). This field strength is consistent with previous work on effective magneto-mechanical biofilm disruption, where the magnetic nanochains actuated at <20 mT and <10 Hz achieved 99.99% bacterial kill when combined with methicillin.^16^

### Protocol optimization: importance of intermediate resuspension

Following magnetic field characterization and before comparing the different AMPs, we optimized the magnetic agitation protocol by assessing whether intermediate manual resuspension between each 3-minute magnetic agitation cycle improved the mechanical disruption of the biofilm. *In situ* visualization of synchronous rotation was recorded at 200 rpm (see the SI, video).

Using MMRs at 100 ppm, three conditions were compared: (i) control without rods (mechanical resuspension only), (ii) MMRs with manual resuspension between each 3-minute cycle, and (iii) MMRs without resuspension (continuous 9-minute agitation). Bacterial detachment from titanium discs was quantified by flow cytometry based on the number of detached bacterial events per µL in the collected suspension. The control conditions produced an average of 707 ± 111 events per µL, whereas agitation with MMRs and intermediate resuspension resulted in 2307 ± 177 events per µL, significantly higher than both the control and the non-resuspension conditions (1550 ± 1111 events per µL, *p* < 0.05, *t*-test). These results confirm that manual resuspension between agitation cycles substantially improves the mechanical efficiency of magnetic actuation by preventing particle aggregation and maintaining homogeneous rotational behavior of the MMRs in suspension. Consequently, the 3 × 3 min RMF protocol with intermediate resuspension was adopted for all subsequent experiments involving other types of smaller AMPs.

### AMP size-dependent mechanisms: microscale detachment *versus* nanoscale membrane damage

**Fig. 3:**
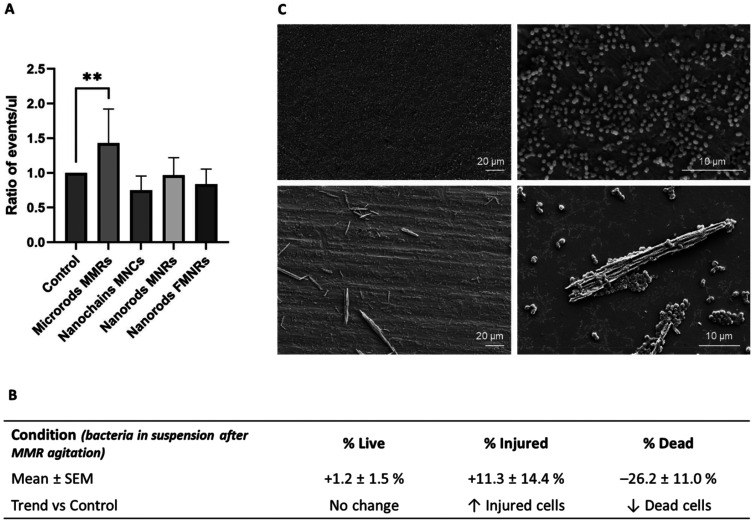
(A) Flow cytometric analysis of *E. faecalis* biofilm detachment from titanium plates after exposure to four different AMP groups and mechanical agitation (3 × 3 min), compared to a non-treated control. The ratio of detached bacteria (events per μL) was significantly increased in the MMR group. The asterisks indicate statistically significant differences between the control and MMR groups (*p* < 0.01, ANOVA with Dunn's multiple comparison test, *α* = 0.005, *n* = 9). (B) Summary table of bacterial populations in suspension after MMR agitation, normalized to the negative control (set to 100%). The values are mean ± SD (*n* = 9). The arrows indicate the direction of change relative to the control (↑ increase and ↓ decrease). These variations represent the general trends and were not statistically significant. (C) SEM images of titanium discs, control (top panels, ×500 and ×2000) and after MMR agitation (bottom panels, ×500 and ×2000), showing reduced surface biofilm coverage and microrod–bacteria interactions.

**Fig. 4:**
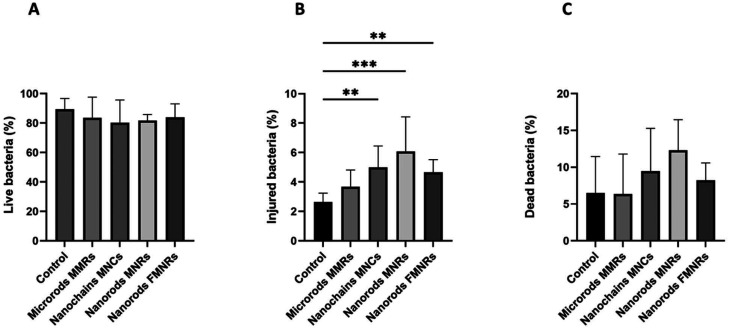
Bacterial viability profiles of *E. faecalis* cells remaining on titanium discs after magnetic agitation with anisotropic rods. Flow cytometry analysis of bacterial viability on the titanium surface after exposure to the MMRs, MNCs, MNRs and FMNRs under a rotating magnetic field (3 × 3 min, 2000 rpm). (A) Percentage of live bacterial cells (SYTO9+/PI−). No significant differences were observed between the treated and control groups. (B) Percentage of injured bacterial cells (SYTO9+/PI+). A significant increase in injured populations was detected for nanoscale rods (MNCs, MNRs, and FMNRs) compared to the control (*p* < 0.005–0.001; ANOVA with Dunn's multiple comparison test, *n* = 9). (C) Percentage of dead bacterial cells (SYTO9−/PI+). Although a slight upward trend was observed across all groups, differences were not statistically significant.

With the optimized RMF agitation protocol established, we next investigated the mechanical and biological effects of the different AMPs on *E. faecalis* biofilms by flow cytometry and viability analysis. Flow cytometry analysis of the impact of mechanical agitation by various magnetic AMPs on 24-hour *E. faecalis* biofilms is presented in Fig. 3 and 4. After magnetic agitation completion using MMRs, a significant increase in the number of detached bacteria from titanium discs was observed (Fig. 3). Specifically, the suspended bacterial population showed a statistically relevant event increase for over 40% in the supernatant compared to the negative control without AMPs (*p* < 0.005) (Fig. 3A). In contrast, no significant increase in detached bacterial load was measured for the other AMPs, including MNCs, MNRs, and FMNRs. To complement the detachment readout (events per µL in the supernatant), SEM imaging was performed on titanium plates to assess the residual surface coverage qualitatively (Fig. 3C). Control samples exhibited a biofilm layer covering the titanium surface, whereas after exposure to MMRs under RMF agitation, exposed titanium areas and microrods in contact with bacterial clusters were clearly observed. In addition, as a simple surface proxy extracted from the representative 2000× SEM fields shown in Fig. 3C, we computed the area fraction occupied by high-contrast biofilm features after background subtraction and automated thresholding (ImageJ; Otsu). This proxy decreased from approximately 20.6% (control) to 9.0% (MMR-treated), supporting a reduction in surface-associated biofilm coverage. We emphasize that this metric is semi-quantitative (representative fields) and does not replace a dedicated biomass staining/3D imaging assay. Control samples exhibited a continuous biofilm layer covering the titanium surface, whereas after exposure to MMRs under RMF agitation, a clear reduction in biofilm surface coverage was observed, with exposed titanium areas and MMRs in direct contact with bacterial clusters. SEM micrographs visually confirm the mechanical removal of biofilm structures and the results of physical interactions between MMRs and the bacterial matrix, while no significant mechanical biofilm detachment was observed for the other types of AMPs. This particle size-dependent detachment can be rationalized through rotational torque considerations. Under an RMF, elongated magnetic particles experience a torque *τ* proportional to their magnetic moment (*μ*) and the applied field (*B*): *τ* = *μ* × *B*. The 12.6 µm length of MMRs generates substantially higher torque compared to sub-micron nanoscale AMPs, due to their higher magnetic moment (larger volume) and also due to their relatively high saturation magnetization (33.9 *vs.* 13–15 emu g^−1^ for MNCs/MNRs). Additionally, the physical dimensions of MMRs (>12 µm) exceed typical bacterial cell dimensions (∼1 µm for *E. faecalis*) and biofilm thickness, enabling them to physically lever apart biofilm structures and shear bacteria from the titanium surface. In contrast, nanoscale AMPs, despite high aspect ratios (up to 19.9 for MNRs), seem to lack the dimension (length) and magnetic moment necessary to generate sufficient mechanical leverage against mature biofilm matrices. This can be due to the dense EPS matrix and strong surface adherence in *E. faecalis* used in our study. The exceptional resistance of *E. faecalis* biofilms to mechanical disruption has been well documented in the literature where it has been shown that it forms more robust biofilms than other *Enterococcus* species,^35^ making this species an ideal stringent model for evaluating magneto-mechanical biofilm removal strategies. In this study, the simultaneous evaluation of microrods, nanorods, and nanochains under identical RMF and biofilm conditions provides a direct and systematic comparison of how size and magnetic characteristics influence biofilm disruption. While it remains challenging to compare the results across studies that use different biofilm models, incubation times, or magnetic field strengths, this internal comparative design uniquely highlights the particle size-dependent transition between microscale detachment and nanoscale membrane-level interactions.

To provide a clinical reference, *E. faecalis* biofilms grown for 24 h on titanium discs were exposed to 0.1% chlorhexidine (CHX) for either 30 s (typical dental clinical exposure) or 9 min, matching the RMF treatment duration. SEM imaging revealed a reduction in biofilm coverage after 30 s and a more pronounced decrease after 9 min. However, residual bacterial structures remained visible on the titanium surface, indicating that CHX treatment under these conditions did not fully eliminate the biofilm (see the SI, Fig. S1). Notably, even extended CHX exposure (9 min) did not fully eliminate the biofilm from the titanium surface, suggesting that chemical disinfection alone may leave residual biofilm structures. Mechanical biofilm disruption strategies may therefore represent a complementary approach for implant surface decontamination. Bacterial viability analysis was also performed after AMP treatment to determine whether bacterial detachment resulted from mechanical action or due to membrane damage and hence biocidal effects. The analysis of the suspended bacteria following MMR treatment revealed that detachment was primarily mechanical and the effect was not biocidal. While the proportion of injured cells (SYTO9+/PI+, indicating partial membrane compromise) showed a non-significant upward trend (+11.3 ± 14.4%), live and dead populations remained relatively unchanged compared to controls (Fig. 3B). Although nanoscale AMPs failed to detach the biofilm, viability analysis of bacteria remaining on titanium surfaces revealed a distinct mechanism: bacterial membrane damage without physical removal (Fig. 4). The proportion of injured cells increased significantly from 2.6 ± 0.8% in controls to 4.9 ± 1.2% (MNCs), 6.1 ± 1.2% (MNRs), and 4.6 ± 1.2% (FMNRs) (*p* < 0.005 to *p* < 0.001; Fig. 4B) and no significant increase was observed for MMRs. Live cell populations remained statistically unchanged (∼83.5%), while dead cells showed non-significant upward trends. Importantly, a copper surface was included as a positive control to assess strong bactericidal activity, which produced marked decreases in live bacteria (33.6% *vs.* 89.5% in the control) and a concomitant increase in injured (20.2% *vs.* 2.6%) and dead cells (45.2% *vs.* 4.4%). This positive control confirmed the sensitivity of the experimental setup to detect strong bactericidal effects.

The nanoscale AMPs induced a slight increase in dead cells and a significant rise in injured cells on the titanium surface compared to the control and MMRs. These injured cells are indicative of stress or sub-lethal damage that can impair bacterial function or enhance susceptibility to adjunctive treatments. These results are consistent with the literature, suggesting that certain nanomaterials can disrupt bacterial membranes without killing the cells, acting through surface interactions or mechanical nano-effects.^36^ Although this study primarily assessed membrane integrity, additional assays could further clarify the physiological stress responses induced by nanoscale AMPs. In particular, evaluating metabolic activity (*e.g.*, NADPH/NADH balance) or stress-related structures such as protein aggregates or stress granules would provide valuable insights into the sublethal effects of magneto-mechanical interactions on bacterial cells.

Numerous studies show that nano-sized magnetic particles (<100 nm) interact more strongly with bacterial membranes than micro-sized particles.^37,38^ Their higher surface-to-volume ratio enhances their ability to attach to and penetrate the bacterial cell envelope, leading to greater membrane disruption, increased permeability, and ultimately cell lysis. In contrast, micro-sized magnetic particles exhibit reduced membrane interaction and lower antibacterial activity, as their larger size limits close contact and penetration into bacterial membranes. Thus, the observed differences between MMRs and other AMPs can be attributed to their respective nanoscale dimensions (*e.g.*, widths of 605, 193, 94, and 10 nm for MMRs, MNCs, MNR, and FMNR, respectively). An additional important factor is the particle number density. All AMP types were applied at identical iron concentrations; therefore, nanoscale AMPs were orders of magnitude more numerous than MMRs. The substantially higher collision/impact frequency between nanoscale particles and bacterial cells likely increases membrane perturbation and explains the higher fraction of membrane-compromised bacteria observed. However, because the magnetic torque scales strongly with particle volume, the torques generated by individual nanoscale particles remain insufficient to overcome biofilm adhesion forces and induce large-scale detachment. Surprisingly, FMNRs with the smallest dimensions compared to other AMPs did not show an increased percentage of injured cells. This phenomenon can be attributed to their ferromagnetic properties creating persistent dipole–dipole attractions that drive the aggregation of FMNRs even without field exposure.^39^ In contrast, superparamagnetic nanoparticles only become magnetized in the presence of an external field and lack remanent magnetization, resulting in much weaker interparticle attractions and less spontaneous aggregation. As a result, the aggregation of FMNRs, especially under a magnetic field, reduces their effective interaction with bacterial membranes, thereby limiting their ability to cause bacterial membrane damage and cell injury.

Importantly, the short-time agitation protocol used in this study, three cycles of 3 minutes, was designed to reflect realistic clinical application in dentistry. In the context of dental biomaterials, particularly in endodontics or implant decontamination, any adjunctive treatment must be both rapid and minimally invasive to be feasible in a dental office setting. Moreover, in our previous study, MNCs showed no toxicity to Caco-2 intestinal epithelial cells, regardless of whether magneto-mechanical force was employed or not.^40^ The chosen protocol ensures that the AMP suspension maintains its physical and colloidal stability during agitation, which is critical since the aggregation of AMPs could reduce mechanical efficacy or alter undesired biological interactions. Maintaining dispersed AMPs is especially important for membrane-level interactions, which were likely responsible for the observed increase in injured cells. Therefore, the current protocol not only demonstrates biological efficacy but also supports potential translational use in chairside treatments, where time efficiency and safety are essential. This may reflect a limitation in treatment duration or intensity, or a need to combine mechanical agitation with chemical or photodynamic agents for enhanced bacteria toxicity, as proposed in earlier work with dentin bonding agents or rose bengal photosensitization.^36,41^

### Practical perspective for implant/dental applications

The two dominant outcomes observed here map onto distinct use cases. First, microrod-driven detachment corresponds to mechanical debridement: a rapid reduction of surface-associated biomass, which is directly relevant for implant-surface decontamination where removal of the EPS-protected structure is the objective. Second, the nanoscale AMPs did not detach the mature biofilm under our short protocol, but increased the SYTO9+/PI+ fraction among surface-associated cells, consistent with increased membrane permeability and sub-lethal stress. Such an effect may be valuable as an adjuvant/sensitization step (*e.g.*, to enhance susceptibility to disinfectants or photodynamic/chemical adjuncts) rather than as a standalone debridement method. These distinctions help define which AMP designs are most appropriate depending on whether the clinical priority is biomass removal or bacterial sensitization under time-constrained chairside protocols.

## Conclusions

This study demonstrates that AMPs exhibit size-dependent mechanisms for biofilm disruption under an RMF. Micron-scale magnetic microrods (MMRs, >12 µm) generate sufficient mechanical torque to physically detach *E. faecalis* biofilms from titanium surfaces, increasing suspended bacterial counts without substantial bactericidal effects. In contrast, nanoscale AMPs (60–880 nm length) fail to detach biofilms due to low magnetic moment but induce significant membrane damage in surface-adherent bacteria, increasing the proportion of membrane-compromised cells. These findings reveal that particle size, rather than magnetic properties (superparamagnetic *vs.* ferromagnetic) or aspect ratio alone, is the dominant design parameter governing the mechanism of action.

## Author contributions

Vincent Pautu: conceptualization, investigation, and writing – original draft; Laurine Marger: conceptualization, investigation and writing – review & editing; Maja Caf: investigation; Fabrice Marger: investigation; Mustapha Mekki: conceptualization and writing – review & editing; Slavko Kralj: conceptualization, writing – review & editing and funding acquisition; and Irena Milošević: conceptualization, writing – review & editing and funding acquisition.

## Conflicts of interest

There are no conflicts to declare.

## Supplementary Material

NR-018-D6NR00046K-s001

NR-018-D6NR00046K-s002

## Data Availability

The supporting data have been provided as part of the supplementary information (SI). Supplementary information: characterization of titanium plates and further experimental procedures are available in Tables S1 and S2, magnetic field characterization in Tables S3 and S4, SEM images before and after CHX treatment in Fig. S1, and *in situ* visualization of synchronous rotation in the SI video. See DOI: https://doi.org/10.1039/d6nr00046k.
