# Supplementary material for: Influence of particle size, shape, and magnetic properties on torque-driven biofilm removal using anisotropic magnetic particles
Source: Nanoscale. 2026 Mar 27;18(19):10203–14. doi: 10.1039/d6nr00046k (PMC13090009; doi:10.1039/d6nr00046k)
Supplement: NR-018-D6NR00046K-s001 [file NR-018-D6NR00046K-s001.pdf]

This journal is © The Royal Society of Chemistry 2026

Supplementary Information

Influence of Particle Size, Shape, and Magnetic Properties on Torque-Driven Biofilm Removal by Anisotropic Magnetic Particles

Vincent Pautu[a], Laurine Marger[b], Maja Caf[c,d], Fabrice Marger[a,b], Mustapha Mekki[b], Slavko Kralj[c,d,]* and Irena Milošević[a]*

[a] *School of Engineering, Architecture and Landscape of Geneva (HEPIA), HES-SO - University of Applied Sciences and Arts Western Switzerland.*
[b] *Laboratory of Biomaterials, Faculty of Medicine, University Clinics of Dental Medicine, University of Geneva, Genève, Switzerland.*
[c] *Department for Materials Synthesis, Jožef Stefan Institute, Jamova 39, Ljubljana, Slovenia.*
[d] *Faculty of Pharmacy, University of Ljubljana, Aškerčeva 7, Ljubljana, Slovenia.*
**irena.milosevic@hesge.ch and slavko.kralj@ijs.si*

Characterization of titanium plates

Profilometry revealed a homogeneous, finely rolled surface texture across all Ti Grade 4 plates. The measured roughness parameters were Ra = 0.824 ± 0.030 µm, Rq = 1.074 ± 0.046 µm, Rc = 4.462 ± 0.320 µm, and Rz = 8.148 ± 1.130 µm (n = 9). Shapiro–Wilk tests supported normality for Ra (p = 0.832), Rq (p = 0.147), and Rz (p = 0.366), while Rc deviated from normality (p = 0.011), consistent with isolated micro-peaks inherent to the rolling process. Overall, these data indicate a stable and isotropic surface topography suitable for reproducible biofilm growth and magneto-mechanical experiments.

Supplementary Table S1: Surface roughness parameters of cold-rolled titanium Grade 4 plates measured by confocal laser profilometry. Values are reported as mean ± standard deviation.

Roughness parameter	Value
Ra (µm)	0.824 ± 0.030
Rq (µm)	1.074 ± 0.046
Rc (µm)	4.462 ± 0.320
Rz (µm)	8.148 ± 1.130
Number of measurements	n = 9

Measurements were performed at five positions per plate using a non-contact confocal laser profilometer. Roughness parameters were calculated according to ISO 4287 and ISO 16610-21.

Static water contact angle and normality assessment

Static water contact angle measurements were performed on cold-rolled titanium Grade 4 plates to characterize surface wettability. For each plate, three independent droplets were deposited at distinct locations, and contact angles were measured 10 s after deposition using Young–Laplace fitting. Across the five titanium plates, a total of 15 individual contact angle values were obtained.

The overall static contact angle was 79.8 ± 0.8° (mean ± standard deviation, n = 5 plates), indicating moderate hydrophilicity consistent with native TiO_2 passivation. Normality of the individual contact angle measurements (n = 15 droplets) was assessed using the Shapiro-Wilk test. The distribution did not significantly deviate from normality (W = 0.958, p = 0.659), supporting the use of parametric descriptive statistics.

Together with the controlled surface roughness, these wettability characteristics define a stable and reproducible surface baseline suitable for subsequent biofilm formation and magneto-mechanical actuation experiments.

Supplementary Table S2: Static water contact angle measurements and normality assessment

Ti plate	Droplet 1 (°)	Droplet 2 (°)	Droplet 3 (°)	Mean ± SD per plate (°)
1	80.30	79.10	79.20	79.53 ± 0.67
2	79.10	79.90	80.80	79.93 ± 0.85
3	80.90	80.30	79.00	80.07 ± 0.97
4	81.20	80.30	79.70	80.40 ± 0.75
5	79.20	78.30	80.10	79.20 ± 0.90
**Overall (plates)**	—	—	—	**79.8 ± 0.8 (n = 5)**
**Normality test (individual droplets, n = 15):** Shapiro–Wilk W = 0.958, p = 0.659				

Contact angles were measured 10 s after droplet deposition using Young–Laplace fitting. Normality testing was performed on individual droplet values (n = 15), while plate-averaged values (n = 5) were used for descriptive reporting.

Magnetic field characterization

The rotating magnetic field generated by the 2mag MIXdrive device was characterized using an axial Hall probe positioned at the bottom center of each well of a 24-well plate. Measurements were performed across all 24 wells for different combinations of rotation speed and power settings in order to document field scaling and plate-level homogeneity.

Across all tested conditions, the magnetic field increased consistently with both rotation speed and power setting. Average magnetic field values and their standard deviations across the 24-well plate are summarized in Supplementary Table S4.

For all conditions, the variation between the lowest and highest field values across the plate remained within approximately 1 to 3 mT, indicating a reasonably homogeneous magnetic field distribution at the scale of the 24-well plate.

All biofilm detachment experiments were performed at 100 % power and 2000 rpm. Under these conditions, the magnetic field at the level of the titanium discs exhibited an average value of 8.60 ± 0.94 mT across the plate. The corresponding well-by-well magnetic field values are reported in Supplementary Table S3.

Supplementary Table S3: Magnetic field values (mT) measured in each well of a 24-well plate at 100 % power and 2000 rpm using the 2mag MIXdrive device. Measurements were performed at the bottom center of each well using an axial Hall probe.

**Field (mT)**	**1**	**2**	**3**	**4**	**5**	**6**
**A**	9.54	8.64	8.80	9.25	7.70	7.20
**B**	9.49	9.56	10.87	8.87	9.01	7.13
**C**	9.49	9.86	9.61	7.96	8.20	7.53
**D**	8.39	7.92	8.32	7.80	7.26	8.04

Supplementary Table S4: Average magnetic field values across the 24-well plate as a function of power setting and rotation speed. Values are reported as mean ± standard deviation across all 24 wells.

Power (%)	Rotation speed (rpm)	Magnetic field (mT)
50	100	0.63 ± 0.04
50	600	2.61 ± 0.21
50	2000	4.02 ± 0.33
100	100	1.54 ± 0.15
100	600	5.91 ± 0.50
100	2000	8.60 ± 0.94

Standard deviations were calculated across the 24 individual well measurements for each condition.

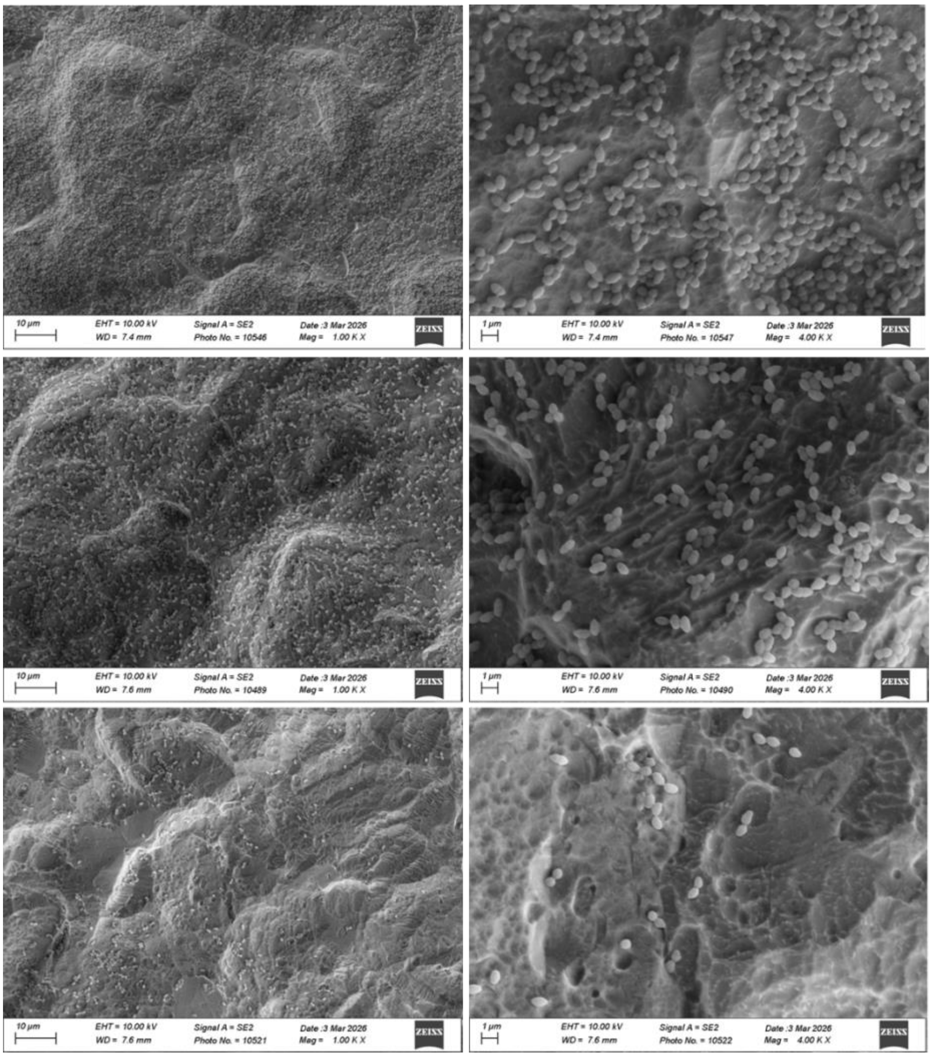

Supplementary Figure S1. SEM images of 24 h *E. faecalis* biofilms grown on titanium discs under control conditions (top row), after treatment with 0.1% chlorhexidine (CHX) for 30 s (middle row), and after 9 min CHX exposure (bottom row). The left column shows images acquired at ×1000 magnification, while the right column presents the corresponding higher magnification views (×4000). A progressive reduction in bacterial surface coverage is observed with increasing CHX exposure time, although residual bacterial structures remain visible on the titanium surface.
